# Diaphragm electromyography results at different high flow nasal cannula flow rates

**DOI:** 10.1007/s00431-019-03401-z

**Published:** 2019-06-11

**Authors:** Eleanor Jeffreys, Katie A Hunt, Theodore Dassios, Anne Greenough

**Affiliations:** 1 Women and Children’s Health, School of Life Course Sciences, Faculty of Life Sciences and Medicine, King’s College London, London, WC2R 2LS UK; 2 Asthma UK Centre in Allergic Mechanisms of Asthma, Kings College London, London, UK; 3 Neonatal Intensive Care Centre, King’s College Hospital NHS Foundation Trust, 4th Floor Golden Jubilee Wing, Denmark Hill, London, SE5 9RS UK; 4 NIHR Biomedical Research Centre at Guy’s and St Thomas’ NHS Foundation Trust and King’s College London, London, UK

**Keywords:** High flow nasal cannula, Diaphragm, Electromyogram

## Abstract

Heated, humidified, high-flow nasal cannula (HHHFNC) is increasingly being used, but there is a paucity of evidence as to the optimum flow rates in prematurely born infants. We have determined the impact of three flow rates on the work of breathing (WOB) assessed by transcutaneous diaphragm electromyography (EMG) amplitude in infants with respiratory distress or bronchopulmonary dysplasia (BPD). Flow rates of 4, 6 and 8 L/min were delivered in random order. The mean amplitude of the EMG trace and mean area under the EMG curve (AEMGC) were calculated and the occurrence of bradycardias and desaturations recorded. Eighteen infants were studied with a median gestational age of 27.8 (range 23.9–33.5) weeks and postnatal age of 54 (range 3–122) days. The median flow rate prior to the study was 5 (range 3–8) L/min and the fraction of inspired oxygen (FiO_2_) was 0.29 (range 0.21–0.50). There were no significant differences between the mean amplitude of the diaphragm EMG and the AEGMC and the number of bradycardias or desaturations between the three flow rates.

*Conclusions*: In infants with respiratory distress or BPD, there was no advantage of using high (8 L/min) compared with lower flow rates (4 or 6 L/min) during support by HHHFNC.

**Table:** 

**What is known:** *• Humidified high flow nasal cannulae (HHHFNC) is increasingly being used as a non-invasive form of respiratory support for prematurely born infants.* *• There is a paucity of evidence regarding the optimum flow rate with 1 to 8 L/min being used.*	
**What is new:** *• We have assessed the work of breathing using the amplitude of the electromyogram of the diaphragm at three HHHFNC flow rates in infants with respiratory distress or BPD.* *• No significant differences were found in the EMG amplitude results or the numbers of bradycardias or desaturations at 4, 6 and 8 L/min.*

**What is known:**

*• Humidified high flow nasal cannulae (HHHFNC) is increasingly being used as a non-invasive form of respiratory support for prematurely born infants.*

*• There is a paucity of evidence regarding the optimum flow rate with 1 to 8 L/min being used.*

**What is new:**

*• We have assessed the work of breathing using the amplitude of the electromyogram of the diaphragm at three HHHFNC flow rates in infants with respiratory distress or BPD.*

*• No significant differences were found in the EMG amplitude results or the numbers of bradycardias or desaturations at 4, 6 and 8 L/min.*

## Introduction

There is increasing use of non-invasive ventilation for prematurely born infants. This includes nasal continuous positive airway pressure (nCPAP) and heated, humidified, high flow nasal cannula (HHHFNC). A systematic review of randomised controlled trials (RCTs) has shown that HHHFNC may be at least an equivalent to nCPAP [20]. A UK survey [16], however, showed that the flow rates used during HHHFNC were variable, from 1 to 8 L/min. Similarly, in Canada [4], starting flow rates ranged from 1 to 6 L/min and maximal flow rates were from 2 to 8 L/min.

There have been studies determining the effect of flow rate on the pressure generated during HHFNC. In an in vitro model, airway pressure was shown to increase with increasing HHFNC flow rates [17], but HHFNC cannula systems could generate uncontrolled CPAP levels and mouth leaks affected the CPAP level [3]. An in vivo study highlighted a linear increase in positive pharyngeal pressure as flow rates during HHHFNC increased from 2 to 8 L/min [21]. Similarly, in another study, a significant association between flows and generated oesophageal pressures was demonstrated, but with variability in the amount of end expiratory pressure generated [5]. Furthermore, a linear relationship between measured pharyngeal pressure and flow rates was seen using either of the two HHFNC devices [1]. A further study demonstrated increasing flow rates led to increasing intrapharyngeal pressures [18]. It might then be expected that increasing flow rates might reduce the work of breathing (WOB). There is, however, a paucity of conflicting evidence regarding how different flow rates of HHHFNC affect the work of breathing. In one study [15], no significant differences in the work of breathing were demonstrated between flow rates of 3, 4, or 5 L/min during HHHFNC and nCPAP delivered at 6 cm H_2_O. In another study, however, compared to a baseline of nCPAP of 6 cm H_2_O, respiratory rates increased when the infants were transferred to HHFNC at 6 L/min and the flow rate was reduced by 1 L/min every 30 min suggesting that WOB had increased [10]. In prematurely born infants, an increased WOB in infants can lead to respiratory acidosis, an increased oxygen requirement and failure to thrive. Thus, it is important to determine if different flow rates during HHFNC do affect the WOB.

There is increased respiratory drive when the work of breathing increases. Neural respiratory drive can be assessed by measuring the electromyogram (EMG) of the diaphragm. The EMG of the diaphragm can now be measured transcutaneously by placing electrodes on the skin overlying the diaphragm [2]. This non-invasive technique has been shown to be well tolerated by infants and yields reproducible results [7].

We hypothesised that at higher flow rates of HHHFNC, there might be higher lung volumes due to the increased pressures and hence a lower WOB and more effective support as assessed by lower levels of desaturations and bradycardias. The aim of this study was to test that hypothesis by determining whether there was a reduction in the work of breathing as assessed by the amplitude of the diaphragm EMG as the flow rate of HHHFNC was increased over the range 4 to 8 L/min in prematurely born infants with respiratory distress or bronchopulmonary dysplasia (BPD). In addition, we wished to determine if higher compared to lower flow rates were associated with fewer bradycardias and desaturations.

## Methods

A randomised cross-over study was conducted at a tertiary neonatal unit in King’s College Hospital NHS Foundation Trust between March and August 2018. The study was approved by the Wales Research Ethics Committee and parents gave written informed consent for their infant to take part. Prematurely born infants of less than or equal to 34 weeks of gestational age who were already on HHHFNC with respiratory distress or BPD were eligible for the study. BPD was diagnosed if the infant was oxygen-dependent for 28 days after birth [6].

HHHFNC was delivered either by an Optiflow device (Fisher and Paykel Healthcare, New Zealand) or an SLE 6000 ventilator (SLE limited, Croydon UK). The nasal prongs (Fisher and Paykel Healthcare, New Zealand) used were chosen such that they occluded less than half the nares, but not the whole of the nares [21]. We did not ensure that the infant’s mouth was closed during the study period as we wished to assess the effect of the different flow rates as they are used in routine clinical practice. The infants received HHHFNC at three flow rates of 4 L/min, 6 L/min and 8 L/min, each for 1 h. The order in which the flow rates were delivered to an infant was determined by a random number generator.

Infants were routinely monitored throughout the study. The FiO_2_ was altered to maintain oxygen saturations between 92 and 96%. Bradycardias were recorded if the heart rate fell below 100 bpm for more than 3 s and a desaturation if the oxygen saturation level (SaO_2_) fell below 88% for more than 3 s. Three seconds was chosen so that no artefactual data were included. The study was stopped if a change in flow rate was not tolerated as evidenced by a greater than 0.1 increase in F_i_O_2_ or there was a bradycardia which lasted more than 10 s or a desaturation for longer than 10 s. Patient characteristics collected included gestational age at birth, postnatal age, birth weight, exposure to antenatal steroids and postnatal surfactant and the pre-study flow rate and FiO_2_.

The transcutaneous diaphragm EMG was monitored using a portable 16-channel digital physiological amplifier (Dipha-16; Inbiolab, Groningen, the Netherlands) and three surface electrodes (Kendall H59P cloth electrodes; Covidien, Massachusetts). The method used to filter the electric activity of the heart was similar to the gating technique described by O’Brien [13]. The device wirelessly transmitted to a bedside computer running Polybench (Applied Biosignals, Weener, Germany). The diaphragm EMG was started at the beginning of the study and recorded continuously throughout the 3 h. Data were analysed from the last 5 min of each hour to allow for acclimatisation of the infant to each new flow rate.

**Fig. 1:**
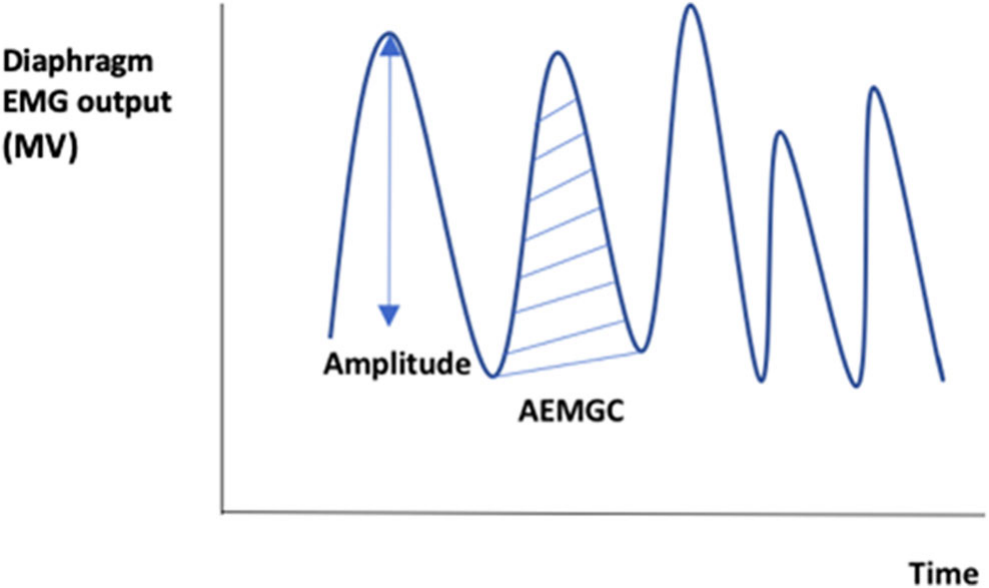
Representation of diaphragm EMG curve showing the amplitude (shown by the vertical line) and area under the curve (AEMGC) (shown by the hatched area)

EMG data were imported into MATLAB from Poly5 format using the TMSi MATLAB Interface (Twente Medical Systems International, Oldenzaal, the Netherlands). A range of time frames for analysis have been used previously ranging from 30 s to 1 min [7, 8]. The first 50 s artefact-free time frame within the last 5 min of each hour was selected, as it was the maximum time frame for all infants without interference. The mean amplitude of the EMG trace (as a measure of the motor unit activity) and the mean area under the EMG curve (AEMGC) as previously described [2] were calculated (Fig. 1) using MATLAB Statistics Toolbox Release 2015b, (The MathWorks, Inc., Natick, MA, USA).

### Analysis

Differences between the three groups were assessed using the Friedman test or chi-square test as appropriate. Post hoc pairwise analysis with Bonferroni correction was carried out if statistically significant differences were found across the three levels. SPSS Statistics Version 24 was used.

### Sample size

Eighteen infants allowed detection between flow rates of one standard deviation in the mean EMG amplitude (1.65 mV, 40% of the AEMGC) with 80% power at the 5% significance level.

## Results

Eighteen infants with a median birthweight of 992 (range 555–2380) g, a median gestational age of 27.8 (range 23.9–33.5) weeks and a median postnatal age of 54 (range 3–122) days were studied. Five had respiratory distress and 13 had BPD. Their median flow rate immediately prior to the study was 5 (range 3–8) L/min and their median F_i_O_2_ was 0.29 (range 0.21–0.50). Thirteen infants had been exposed antenatally to corticosteroids and 17 had been given surfactant postnatally. In none of the infants was the study stopped prematurely.

**Fig. 2:**
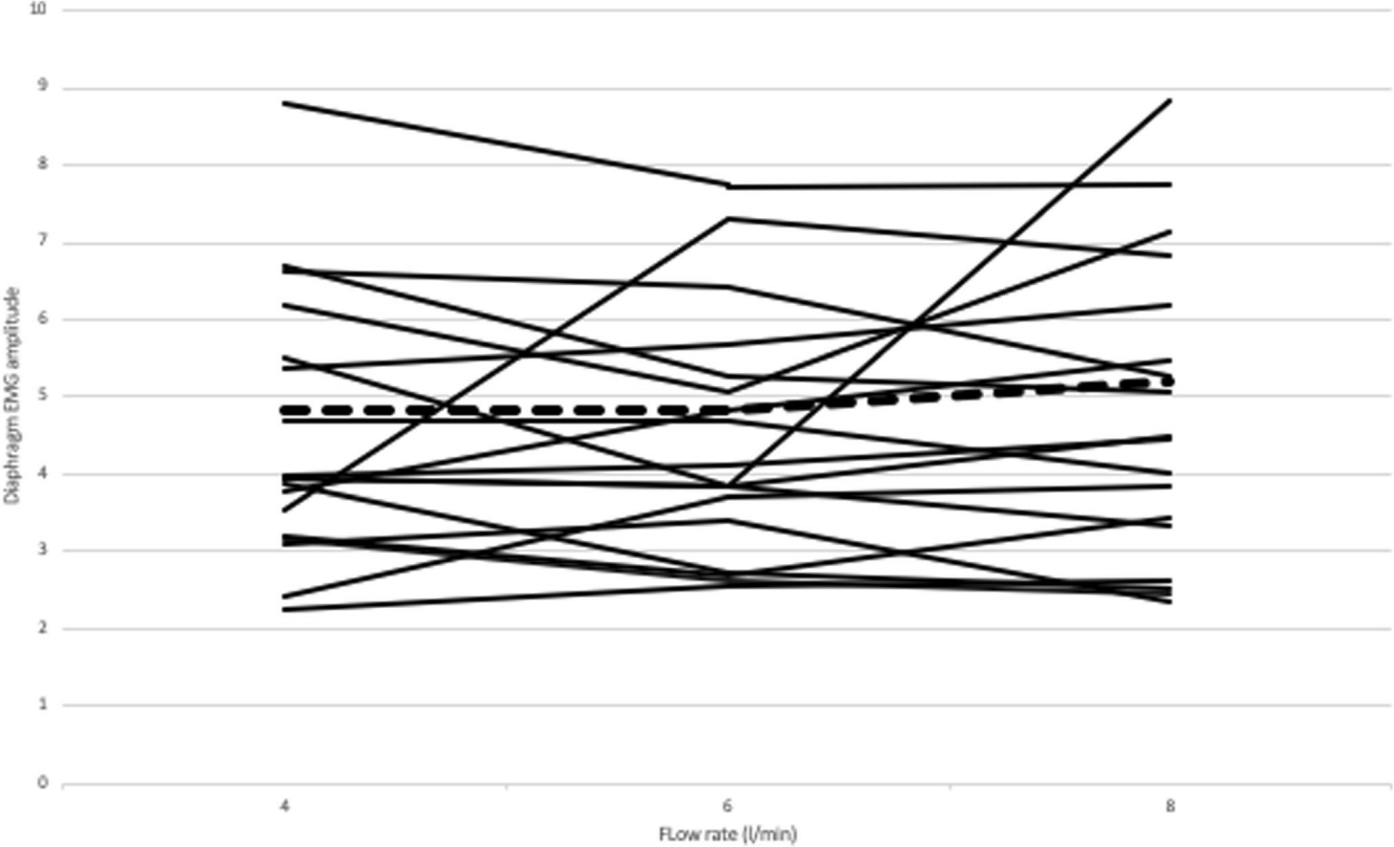
Diaphragm EMG amplitude plotted against the three flow rates. Each individual’s data points are linked and the average of all the infants’ data shown by the dashed line

There were no significant differences between the diaphragm EMG amplitude (Fig. 2) (*p* = 0.678) or the AEGMC (*p* = 0.946) between the three flow rates (Table 1). Equally, the lowest EMG amplitude experienced at the different flow rates did not differ significantly according to levels being in six infants at 4 L/min, five at 6 L/min and seven at 8 L/min (*p* = 0.778) and the smallest AEMGC was experienced by five infants at 4 L/min, five at 6 L/min and eight at 8 L/min (*p* = 0.472). There were also no significant differences in the number of bradycardias (*p* = 0.368) or desaturations (*p* = 0.276) between the three flow rates (Table 1).

**Table 1:** Diaphragm EMG amplitude, AEMGC, desaturations and bradycardias at the three flow rates. Data are displayed as median (range) or n

	4 L/min	6 L/min	8 L/min
Amplitude (mV)	3.96 (2.25–8.80)	4.00 (2.57–7.75)	4.47 (2.36–8.82)
AEMGC (mVs)	1.62 (1.15–3.44)	1.74 (1.15–3.48	1.86 (1.06–3.74)
Desaturations	8	6	5
Bradycardias	1	1	0

## Discussion

We have demonstrated that there were no significant differences in the diaphragmatic EMG amplitude between three flow rates during HHHFNC, nor did we see any significant differences in the proportions of infants with the lowest EMG amplitudes or AEGMC between the three different flow rates. Furthermore, we also assessed the occurrence of bradycardias or desaturations and found no significant differences at the three flow rates. Our findings are consistent with that of Saslow et al. [15] who showed no change in distending pressure between three flow rates of HHHFNC, but they used a smaller range of flow rates of three to 5 L/min.

The lack of significant differences between our three groups may reflect the variable pressure delivered during HHHFNC. Although, in one study, a linear increase in pharyngeal pressure as flow rate increased was demonstrated, independent of whether the mouth was open or closed; in another [9], no positive pressure was generated while the oral cavity was open. We did not ensure the infant’s mouth was closed during the study as we wished to examine the effect of different flow rates as used in routine clinical practice and hence our results would be generalizable.

There are strengths and some limitations to our study. Three flow rates were assessed in each infant thus, although the infants recruited had a wide range of postnatal ages, they each acted as their own controls. The median FiO_2_ immediately before recruitment to the study suggested that some of the infants had mild disease, but there was a range of FiO_2_ levels. Indeed, the infants were typical of those on our NICU who receive HHHFNC and similar to those on other units [16]. We have included a figure of the individual data which demonstrates there was no obvious trend according to the results according to flow rate amongst infants with respiratory distress of evolving or established BPD. Differences in skin contact and anatomical differences between infants can cause variability in the results obtained, yet as seen in Fig. 2, the majority of infants resulted in the same manner to the three flow levels. We have not included in our study infants with very severe respiratory failure, as in our experience, such infants are not routinely supported by HHFNC. We did not measure CO_2_ levels as the infants did not have indwelling arterial lines. We recorded FiO_2_ changes as highlighted by the number of desaturations and did not show any significant differences according to the flow rate. The diaphragmatic EMG was assessed transcutaneously as previously reported [2]. This technique only assesses the electrical activity of the frontal diaphragm and not the intercostal muscles [12]. In preterm infants, however, the intercostal muscles are not thought to have a substantial contribution to breathing effort during tidal breathing [19]. Furthermore, this technique has demonstrated that weaning from CPAP to low flow nasal cannula led to an increase in diaphragmatic activity measured by EMG and was most prominent in preterm infants who failed the weaning attempt [7]. We assessed the diaphragmatic EMG which acts as a surrogate of the work of breathing and has been shown to differ between infants receiving no respiratory support and those on HHHFNC at 6 L/min [14]. The amplitude of the diaphragm EMG, however, may not be fully representative of the work of breathing, but in addition to assessing the diaphragm EMG, we also examined the occurrence of desaturations and bradycardias and demonstrated no significant differences in those outcomes between the three flow rates. Our results are consistent with those of de Waal et al. [2] who suggested that different HHHFNC flow rates in stable infants do not influence work of breathing as assessed by the neural respiratory drive.

In conclusion, we have demonstrated that in prematurely born infants with respiratory distress or BPD, there were no statistically significant differences in the amplitude of the diaphragm EMG or the numbers of desaturations or bradycardias at three different levels of HHHFNC. We have not undertaken a sub-analysis according to underlying diagnosis, as our sample size highlights such a sub-analysis would be underpowered. Our individual results, however, show similar results in all infants. These results suggest there may be no overall advantages of using high compared to lower flow rates. We suggest practitioners should individualise the HHHFNC level taking into account the severity of the infants’ lung disease using the FiO_2_ level and objectively assess the effect of different flow rates by recording FiO_2_ changes and the number of desaturations and bradycardias.

## Abbreviations

AEMGC: Area under the EMG curve
BPD: Bronchopulmonary dysplasia
EMG: Electromyogram
FiO_2_: Fraction of inspired oxygen
HHHFNC: Heated, humidified, high-flow nasal cannula
nCPAP: Nasal continuous positive airway pressure
RCT: Randomised controlled trial
SaO_2_: Oxygen saturation level
